# A soft-computation hybrid method for search of the antibiotic-resistant gene in *Mycobacterium tuberculosis* for promising drug target identification and antimycobacterial lead discovery

**DOI:** 10.1093/bioadv/vbad090

**Published:** 2023-07-27

**Authors:** Neha Jaiswal, Awanish Kumar

**Affiliations:** Department of Biotechnology, National Institute of Technology, Raipur, Chhattisgarh 492010, India; Department of Biotechnology, National Institute of Technology, Raipur, Chhattisgarh 492010, India

## Abstract

Tuberculosis (TB) control programs were already piloted before the COVID-19 pandemic commenced and the global TB response was amplified by the pandemic. To combat the global TB epidemic, drug repurposing, novel drug discovery, identification and targeting of the antimicrobial resistance (AMR) genes, and addressing social determinants of TB are required. The study aimed to identify AMR genes in *Mycobacterium tuberculosis* (MTB) and a new anti-mycobacterial drug candidate. In this research, we used a few software to explore some AMR genes as a target protein in MTB and identified some potent antimycobacterial agents. We used Maestro v12.8 software, along with STRING v11.0, KEGG and Pass Server databases to gain a deeper understanding of MTB AMR genes as drug targets. Computer-aided analysis was used to identify mtrA and katG AMR genes as potential drug targets to depict some antimycobacterial drug candidates. Based on docking scores of –4.218 and –6.161, carvacrol was identified as a potent inhibitor against both drug targets. This research offers drug target identification and discovery of antimycobacterial leads, a unique and promising approach to combating the challenge of antibiotic resistance in *Mycobacterium*, and contributes to the development of a potential futuristic solution.

## 1 Introduction

*Mycobacterium tuberculosis* (MTB) is the bacterium responsible for causing Tuberculosis (TB), which is considered as world's most lethal infectious disease agent, particularly in developing countries such as South-East Asia, India, Africa, Western Pacific, Indonesia, China, Eastern Mediterranean, Philippines, Pakistan, Nigeria, Bangladesh, South Africa, America and Europe (Chakaya *et al.*, 2021). This bacterium spreads rapidly in crowded areas and exacerbates the transmission (Pereira *et al.*, 2005). TB primarily affects the respiratory system and symptoms include persistent coughing, chest discomfort, coughing up blood, weakness, weight loss, fever and night sweats (Zaman, 2010). The World Health Organization (WHO) has been publishing an annual report on TB prevalence since 1996, which represents over 99% of the global population from 198 countries and territories (Chakaya *et al.*, 2021). In 2019, there are approximately 10.0 million people worldwide who are suffering from TB, leading to 1.2 million deaths among HIV-negative individuals and 208 000 deaths among HIV-positive individuals (Zaman, 2010). According to recent estimates, approximately 1.7 billion people worldwide are assessed to be infected with MTB. Based on the estimates of latently infected people by TB, it will cause 16.3 and 8.3 active tuberculosis cases for every 100 000 people in 2030 and 2050 (Chakaya *et al.*, 2021). Globally, over half a million people have been affected by rifampicin-resistant TB, while 78% have been affected by multidrug-resistant (MDR) TB, according to the WHO report (https://www.who.int/teams/global-tuberculosis-programme/tb-reports/global-tuberculosis-report2022/tb-disease-burden/2-3-drug-resistant-tb) (Chakaya *et al.*, 2022). MDR strains are resistant to anti-tuberculosis drugs like isoniazid (Inh), rifampicin (Rif), ethambutol (E) and pyrazinamide; whereas extensive drug-resistant (XDR) strains are resistant to injectable second-line anti-tuberculosis drugs like amikacin, kanamycin and capreomycin (Casadevall, 2017; Pontali *et al.*, 2019). A study by Ballell *et al.* (2005) found that MTB prevalence and deaths are increasing exponentially despite efficient first-line drugs.

TB remains a significant global health problem, with a high burden in many parts of the world. According to the latest data, the highest prevalence of TB is in the South-East Asia region, which accounts for 44% of the total cases worldwide. India also has a high burden, with 26% of the cases, while Africa reports 25% of the global TB burden. The Western Pacific region follows, contributing 18% of the cases, with Indonesia at 8.5% and China at 8.4%. The Eastern Mediterranean region reports 8.2% of the cases, with the Philippines at 6%, Pakistan at 5% and Nigeria at 4.4%. Notably, both Bangladesh and South Africa have a high burden of 3.6%. The American region reports 2.9% of the cases, while Europe has the lowest burden at 2.5% (Chakaya *et al.*, 2021). Therefore, TB therapy today requires the use of several bactericidal and sterilizing drugs over long periods to eradicate active MTB, while simultaneously inhibiting the development of antibiotic resistance in existing bacteria (Ma *et al.*, 2020; Nathanson *et al.*, 1977). Consequently, it is necessary to develop new therapeutic agents, which will be effective against MDR and XDR bacteria without causing adverse effects during long-term use in the long run.

Throughout the world, plants are being used to treat a wide range of diseases because they have a large number of bioactive metabolites. Plant extracts have been proven to have immunomodulatory potentials in a variety of cell lines and animal models, apart from their anti-tuberculosis capabilities. It is estimated that approximately 75% of recognized anti-infective drugs are derived from medicinal plants because they are comparatively less toxic and safe (Cragg *et al.*, 1997). Currently, more than 350 plant species have been investigated for their potential applications in treating tuberculosis in traditional medicine (Eldeen and van Staden, 2007). Phenolic compounds are one of the major classes that can be used in treating many diseases. Plant-derived phenolics can inhibit the growth and activity of many microbes, including many clinically important bacteria, fungi, protozoa and viruses. Therefore, we have selected the phenolic compounds to understand their antimicrobial efficacy against MTB in this study. According to research, phenolic compounds are promising inhibitors and drug candidates for TB and over the past 17 years, phenolic compounds are found to be potent candidates in the treatment of MTB (Kumar *et al.*, 2022; Mazlun *et al.*, 2019; Tyring, 2012). There are a variety of plants such as *Phyllanthus embilica, Bauhinia racemose* and others that produce such secondary metabolites (Prabhu *et al.*, 2017, 2021). In addition, anticancer drugs such as Taxol and Vinblastine are derived from *Catharanthus roseus* and *Taxus brevifolia*, a traditional Chinese medicinal plant (Veeresham, 2012). An estimated 25% of all FDA-approved drugs over the past 20 years have been based on natural products or their derivatives, according to a review published in 2018 (Patridge *et al.*, 2016; Thomford *et al.*, 2018). Furthermore, 65% of the world's population uses organic herbal formulations to treat stomach pain, poison bite, dengue, Corona and respiratory disorders, including *Caricca papaya*, *Andrographis paniculata* and *Calotropis procera* (Farnsworth *et al.*, 1985).

The use of natural products has been successfully integrated into the healthcare system because of its pharmacological potential and it is also estimated that 95% of public hospitals in China have traditional medicine departments. In the search for medicine from different classes of natural molecules, the pharmacological significance of natural phenolic compounds against other antimicrobial diseases is quite experienced^18^; however, their antimycobacterial effect is still unfamiliar to us. Therefore, we used computational analysis to investigate phenolic compounds' antimycobacterial potential and develop effective antimycobacterial pharmaceutical candidates in the future. We propose a hybrid novel approach that combines data mining and molecular docking to identify MTB AMR genes as a drug target and phenolic compounds as potential inhibitors of TB.

## 2 Methods

### 2.1 Identification of AMR genes

To identify AMR genes, present in *M.tuberculosis*, CARD (Comprehensive Antibiotic Resistance Database) (Chen *et al.*, 2020) has been used as a resistance gene identifier. CARD (https://card.mcmaster.ca/ontology/39867) provides quick information about AMR genes, and their potential, and correlates with the actual resistance trait of bacteria (Camiade *et al.*, 2020; Hendriksen *et al.*, 2019; Thomas *et al.*, 2017). Gene family, drug class, resistance mechanism and protein homology have been analyzed and AMR genes of MTB were shortlisted.

### 2.2 Search of MTB AMR gene in the human genome as non-homologous gene

The FASTA sequences of shortlisted AMR genes were downloaded using the Uniprot website by searching for the gene name through the search box at https://www.uniprot.org/. Gene sequences have been analyzed for their homology concerning the human genome using Basic Local Alignment Search Tool (BLAST).

### 2.3 Target determination and subcellular localization of selected MTB AMR genes

AMR has played a noteworthy role in the number of human deaths due to TB in particular (Castro *et al.*, 2021); hence, they are a promising target in drug development. To be considered a drug target, proteins must be important to the pathogen's survival within a host's body, as well as not being homologous to those of the host. The non-homologous AMR genes were selected to prevent the cross-binding of the drug to the host protein, which would result in a greater probability of adverse effects (Sakharkar *et al.*, 2004). Localization and protein family are the major parameters for drug target analysis. The AMR gene sequences were obtained from https://card.mcmaster.ca/ontology/39867. CELLO v.2.5: subCELlular LOcalization predictor tool was used to determine a gene's subcellular location. A query protein sequence (FASTA format) has been submitted to http://cello.life.nctu.edu.tw/. Based on the analysis report, localization, reliability parameters and functional protein family were identified using PFA (protein family analysis). As part of the query box and website https://pfam.xfam.org/search/sequence, the query protein FATSA sequence has been inserted.

### 2.4 Functional process and pathway analysis of MTB AMR genes

The metabolic pathway information has been retrieved from the Kyoto Encyclopedia of Genes and Genomes (KEGG) database (Kanehisa *et al.*, 2022). The assigned ID of *M.tuberculosis* and gene name has been retrieved via https://www.genome.jp/kegg/pathway.html. KEGG is a broad-spectrum source of metabolic pathways information that helps to identify unique proteins and to explore metabolic pathways and their respective protein sequence.

### 2.5 Protein–protein interaction

Protein–protein interaction was predicted using STRING, an online server tool that integrates both known and predicted protein–protein interactions (Szklarczyk *et al.*, 2021). To seek potential interaction between our AMR genes, the STRING tool has been employed by putting gene names and organism names on the query site in https://STRING-db.org/cgi/input?sessionId=biiK4a6BFLeg&input_page_show_search=on. For active interactions, scores > 0.4 were applied and Cytoscape software version 3.6.1 has been used to visualize the PPI networks.

### 2.6 Antigenicity and allergenicity evolution of AMR target proteins

The VexiJen v2.0 software was used to predict the antigenicity and allergenicity of the AMR proteins. The FASTA sequences of individual genes were used as query sequences and sequences with a score over 0.4 are considered antigenic proteins. Allergenicity and antigenicity play important roles in disease diagnosis, the target protein can be antigen but not allergen (Zhang and Tao, 2015).

### 2.7 Collection and categorization of phenolic compounds from a natural source and their antimycobacterial activity analysis

Based on a literature survey, phenolic compounds were identified and classified using PubChem. The anti-mycobacterial activity was assessed using Prediction of Activity Spectra for Substances (PASS) online. PASS Online (http://www.way2drug.com/passonline/predict.php) is a web-based tool to predict the biological activity of compounds. It utilizes computational models and algorithms to analyze chemical structures and predict their activity profiles. Compounds with an antimycobacterial activity score of more than 0.5 were selected for docking analysis.

### 2.8 Natural phenolics–target protein interaction analysis

As part of the docking analysis of phenolic–target protein interactions, Schrodinger Maestro 12.8 software has been utilized. The phenolic compounds have been shortlisted based on their antimycobacterial activity and 5 compounds were selected for docking analysis.

#### 2.8.1 Ligand preparation

To obtain optimized structures, five selected phenolic compounds were prepared for ligand preparation using a 2D sketcher workspace on Schrodinger Maestro 12.8.

#### 2.8.2 Macromolecule/protein selection and preparation

Protein Data Bank (PDB) is the global archive for accessing the 3D structures of biological macromolecules, as it contains information about the majority of proteins efficiently and instantly (Burley *et al.*, 2017). The crystal structure of our selected target proteins was retrieved from PDB (https://www.rcsb.org/) in PDB format. Only two AMR proteins (mtrA and katG) have PDB structures, so the selected compounds were docked with these two proteins to determine their docking potential and hydrogen bond interactions. For protein preparation, the task protein preparation wizard has been used in Schrodinger Maestro 12.8 software. All water molecules and ligands present in proteins were removed and the active binding site has been generated.

#### 2.8.3 Site mapping and grid generation

A molecular docking technique involves finding a ligand-binding region on a protein. The active site of target proteins has been generated after protein preparation. The sitemap in Maestro 12.8 has been used and grid sites were generated for ligand docking.

#### 2.8.4 Molecular docking

For depth, analysis of intermolecular interaction and binding mode between the target proteins of MTB and plant phenolic compounds, the molecular docking analysis studied was executed for selected ligands using the Maestro 12.8 software. The interactions between active sites in the target protein and ligand include the type of interaction and bond distances.

### 2.9 ADMET profiling

ADMET profiling is one of the most important tools in early drug discovery for the identification of active lead compounds(Tsaioun *et al.*, 2009). The ADMETlab2.0 and admetSAR tools were used to evaluate the pharmacokinetic properties of the top phytochemicals(Cheng *et al.*, 2012; Xiong *et al.*, 2021). Absorption, metabolism, distribution, excretion and toxicity (ADMET) are pharmacokinetic properties of a drug accessed in the body.

## 3 Results

The AMR genes which were non-homologous to the human genome were selected and their subcellular localization, functional family and pathways involved were identified. The selection of target genes was done by protein–protein interaction analysis and their antigenicity evaluation. Then we evaluated the phenolic compounds by their antimycobacterial activity identification, the phenolic compounds were shortlisted based on their activity score (more than 0.5). The intermolecular interaction (phenolic compounds and target proteins from the AMR list) was done using molecular docking analysis.

### 3.1 Identification and selection of AMR genes

In this study, the CARD analysis tool was used to analyze the AMR genes of MTB. 10 strict hit genes were observed in the MTB genome as shown in Table 1. These genes contribute to AMR via efflux mechanism and antibiotic target protection, alteration and inactivation. The efflux mechanism is classified into major facilitator superfamily (MFS) and resistance nodulation cell division (RND). The antibiotic target protection/alteration/inactivation was classified according to different classes like antibiotic resistance (quinolone, isoniazid, rifamycin) and gene mutation (23S rRNA with mutation conferring resistance to macrolide antibiotics, Erm 23S ribosomal RNA methyltransferase, murA transferase). These changes in genes confer that AMR was achieved through detoxification, which involves the export of a variety of toxic compounds outside of the cell adaptation of the host cell environment and temperature. Furthermore, the AMR genes present in MTB can be structurally homologous to the human genome. Therefore, we performed BLAST of MTB AMR genes with the human genome to identify sequence homology. All AMR genes were non-homologous except efpA and rpoB genes as shown in Table 2. The non-homologous AMR genes in humans were selected for further analysis.

**Table 1.** AMR gene in *Mycobacterium tuberculosis* as per CARD analysis

AMR gene	Accession no.	Detection criteria	AMR gene family	Drug class	Resistance mechanism	% Match
MfpA (pentapeptide repeat protein MfpA**)**	ARO:3003035	Protein homolog model	Quinolone resistance protein (qnr)	Fluoroquinolone antibiotic	Antibiotic target protection	100.0
EfpA (uncharacterized MFS-type transporter EfpA)	ARO:3003955	Protein homolog model	Major facilitator superfamily (MFS) antibiotic efflux pump	Fosfomycin, cephalosporin, nucleoside antibiotic, glycylcycline, peptide antibiotic, benzalkonium chloride, lincosamide antibiotic, isoniazid, macrolide antibiotic, tetracycline antibiotic, nitroimidazole antibiotic, oxazolidinone antibiotic, acridine dye, rifamycin antibiotic, rhodamine, fluoroquinolone antibiotic, antibacterial free fatty acids, diaminopyrimidine antibiotic, penam, bicyclomycin, phenicol antibiotic	Antibiotic efflux	100.0
AAC (2′)-Ic (aminoglycoside 2′-N-acetyltransferase)	ARO:3002525	Protein homolog model	AAC (2′)	Aminoglycoside antibiotic	Antibiotic inactivation	100.0
MtrA (DNA-binding response regulator MtrA)	ARO:3000816	Protein homolog model	Resistance-nodulation-cell division (RND) antibiotic efflux pump	Antibacterial free fatty acids, tetracycline antibiotic, acridine dye, fluoroquinolone antibiotic, aminocoumarin antibiotic, glycylcycline, macrolide antibiotic, monobactam, aminoglycoside antibiotic, diaminopyrimidine antibiotic, carbapenem, phenicol antibiotic, penam, triclosan	Antibiotic efflux	100.0
*Mycobacterium avium* 23S rRNA with mutation conferring resistance to clarithromycin	ARO:3004164	rRNA gene variant model	23S rRNA with mutation conferring resistance to macrolide antibiotics	Pleuromutilin antibiotic, lincosamide antibiotic, streptogramin antibiotic, glycopeptide antibiotic, macrolide antibiotic, phenicol antibiotic	Antibiotic target alteration	95.38
RbpA (RNA polymerase-binding protein RbpA)	ARO:3000245	Protein homolog model	RbpA bacterial RNA polymerase-binding protein	Rifamycin antibiotic	Antibiotic target protection	91.89
Erm (37) (23S rRNA (Adenine (2058)-N (6))-methyltransferase Erm (37))	ARO:3000392	Protein homolog model	Erm 23S ribosomal RNA methyltransferase	Lincosamide antibiotic, streptogramin antibiotic, macrolide antibiotic	Antibiotic target alteration	99.33
*Mycobacterium tuberculosis* intrinsic murA conferring resistance to fosfomycin (UDP-N-acetylglucosamine 1-carboxyvinyltransferase)	ARO:3003784	Protein variant model	murA transferase	Fosfomycin	Antibiotic target alteration	100.0
*Mycobacterium tuberculosis* katG mutations conferring resistance to isoniazid (Catalase-peroxidase)	ARO:3003392	Protein variant model	Isoniazid resistant katG	Isoniazid	Antibiotic target alteration	100.0
*Mycobacterium tuberculosis* rpoB mutants conferring resistance to rifampicin (DNA-directed RNA polymerase subunit beta)	ARO:3003283	Protein variant model	Rifamycin-resistant beta-subunit of RNA polymerase (rpoB)	Peptide antibiotic, rifamycin antibiotic	Antibiotic target alteration, antibiotic target replacement	100.0

**Table 2.** Sequence homology, subcellular localization, protein family and function of MTB AMR genes

Antibiotic resistance gene of MTB	Non-homologue to human	Subcellular localization	Function
mfpA	Non-homologous	Cytoplasmic	Might be involved in fluoroquinolone resistance (PubMed:15933203). Inhibits ATP-independent DNA relaxation, ATP-dependent DNA supercoiling and ATP-dependent decatenation by endogenous gyrase, 50% inhibition occurs at 2 µM; inhibition is abolished if GyrA is mutated (Asp-87 to Gly or His) (PubMed:19060136). Also inhibits fluoroquinolone-promoted dsDNA cleavage (PubMed:19060136). Increases fluoroquinolone (ciprofloxacin or moxifloxacin) inhibition of gyrase supercoiling activity in a concentration-dependent manner (PubMed:19060136). Inhibits DNA relaxation and supercoiling by *E.coli* gyrase (PubMed:15933203). Forms a structure that exhibits size, shape and electrostatic similarity to B-form DNA; it may bind to DNA gyrase which is postulated to protect it from fluoroquinolones (PubMed:15933203).
efpA	Homologous	Membrane	Transmembrane transporter activity
AAC (2')-Ic	Non-homologous	Plasma membrane	May catalyze the coenzyme A-dependent acetylation of the 2' hydroxyl or amino group of a broad spectrum of aminoglycosides and confer resistance to aminoglycosides (By similarity). In vitro assays show no significant increase of resistance to aminoglycosides, possibly due to low expression in a heterologous system (PubMed:9159528).
mtrA	Non-homologous	Cytoplasm	Member of the two-component regulatory system MtrA/MtrB. Binds direct repeat motifs of sequence 5′-GTCACAGCG-3′, phosphorylation confers higher affinity. Overexpression decreases bacteria viability upon infection of human THP-1 macrophage cell line, due at least in part to impaired blockage of phagosome-lysosome fusion (upon infection bacteria usually remain in phagosomes). Infecting C57BL/6 mice with an overexpressing strain leads to an attentuated infection in both spleen and lungs. The level of dnaA mRNA increases dramatically. Binds the promoter of dnaA, fbpD, ripA and itself, as well as oriC, which it may regulate. Upon co-overexpression of MrtA and MtrB growth in macrophages is partially restored, dnaA expression is not induced, although mouse infections are still attenuated, suggesting that bacterial growth in macrophages requires an optimal ratio of MtrB to MtrA.
*Mycobacterium avium* 23S rRNA with mutation conferring resistance to clarithromycin	Non-homologous	Cytoplasm	Specifically methylates position 2 of adenine 2503 in 23S rRNA and position 2 of adenine 37 in tRNAs.
RbpA	Non-homologous		Binds to RNA polymerase (RNAP), stimulating transcription from principal, but not alternative sigma factor promoters.
Erm (37)	Non-homologous		Transferase
*Mycobacterium tuberculosis* intrinsic murA conferring resistance to fosfomycin	Non-homologous	Cytoplasm	Cell wall formation. Adds enolpyruvyl to UDP-N-acetylglucosamine.
*Mycobacterium tuberculosis* katG mutations conferring resistance to isoniazid	Non-homologous	Cell Wall Cytosol Extracellular region or secreted Plasma Membrane	Bifunctional enzyme with both catalase and broad-spectrum peroxidase activity, oxidizing various electron donors including NADP (H) (PubMed:9006925, PubMed:18178143). Protects *M.tuberculosis* against toxic reactive oxygen species (ROS) including hydrogen peroxide as well as organic peroxides and thus contributes to its survival within host macrophages by countering the phagocyte oxidative burst (PubMed:8658136, PubMed:15165233). Also displays efficient peroxynitritase activity, which may help the bacterium to persist in macrophages (PubMed:10080924). UniRule annotation5 Publications Might be involved in DNA repair. Partly complements recA-deficient *E.coli* cells exposed to UV radiation, mitomycin C or hydrogen peroxide. Increases resistance to mitomycin C in *E.coli* cells deficient for either uvrA, uvrB or uvrC. 1 Publication Catalyzes the oxidative activation of the antitubercular pro-drug isoniazid (INH) to generate an isonicotinoyl radical that then reacts nonenzymatically with NAD to form an isonicotinoyl-NAD adduct which inhibits InhA.
*Mycobacterium tuberculosis* rpoB mutants conferring resistance to rifampicin	Homologous	Cell wall Cytosol Plasma Membrane	DNA-dependent RNA polymerase catalyzes the transcription of DNA into RNA using the four ribonucleoside triphosphates as substrates.

### 3.2 Navigating the subcellular localization, protein function and pathways analysis

Localization of target protein becomes important since proteins can be found in many different locations of the cell. Further, protein function was also analyzed. According to the result, mfpA and mtrA confer resistance to clarithromycin. *Mycobacterium tuberculosis* intrinsic murA conferring resistance to fosfomycin and *M. tuberculosis* katG mutations conferring resistance to isoniazid and they are identified as cytoplasmic proteins (Table 2). Cytoplasmic proteins have multiple functional roles in drug resistance mechanisms (Table 2) and they are considered favorable drug targets. The molecular pathways of these AMR genes were identified and they are involved in drug metabolisms, transportation and biosynthesis pathways as shown in Table 3.

**Table 3.** KEGG pathway analysis, antigenicity and allergenicity prediction of AMR genes of MBT

Antibiotic resistance gene of MTB	Pathways involved	Probability of antigenicity^a^	Allergenicity	Remarks
MfpA	NM	0.6587	Non-Allergen	Antigen
AAC (2′)-Ic	NM	0.4555	Non-Allergen	Antigen
MtrA	mtu00790 Folate biosynthesis mtu01100 Metabolic pathways mtu01240 Biosynthesis of cofactors mtu02020 Two-component system	0.5875	Non-Allergen	Antigen
*Mycobacterium avium* 23S rRNA with mutation conferring resistance to clarithromycin	NM	0.5112	Non-Allergen	Antigen
RbpA	NM	0.3816	Non-Allergen	Non-antigen
Erm (37)	mtu02010 ABC transporters	0.4398	Non-Allergen	Antigen
*Mycobacterium tuberculosis* intrinsic murA conferring resistance to fosfomycin	mtu00520 Amino sugar and nucleotide sugar metabolism mtu00550 Peptidoglycan biosynthesis mtu01100 Metabolic pathways mtu01250 Biosynthesis of nucleotide sugars	0.5485	Allergen	Antigen
*Mycobacterium tuberculosis* katG mutations conferring resistance to isoniazid	mtu00360 Phenylalanine metabolism mtu00380 Tryptophan metabolism mtu00983 Drug metabolism—other enzymes mtu01100 Metabolic pathways mtu01110 Biosynthesis of secondary metabolites	0.5709	Non-Allergen	Antigen

*Note*: NM, not mentioned.

a Value ≥ 0.4 is recommended as antigenic.

### 3.3 Network construction and target protein analysis

**Figure 1.**
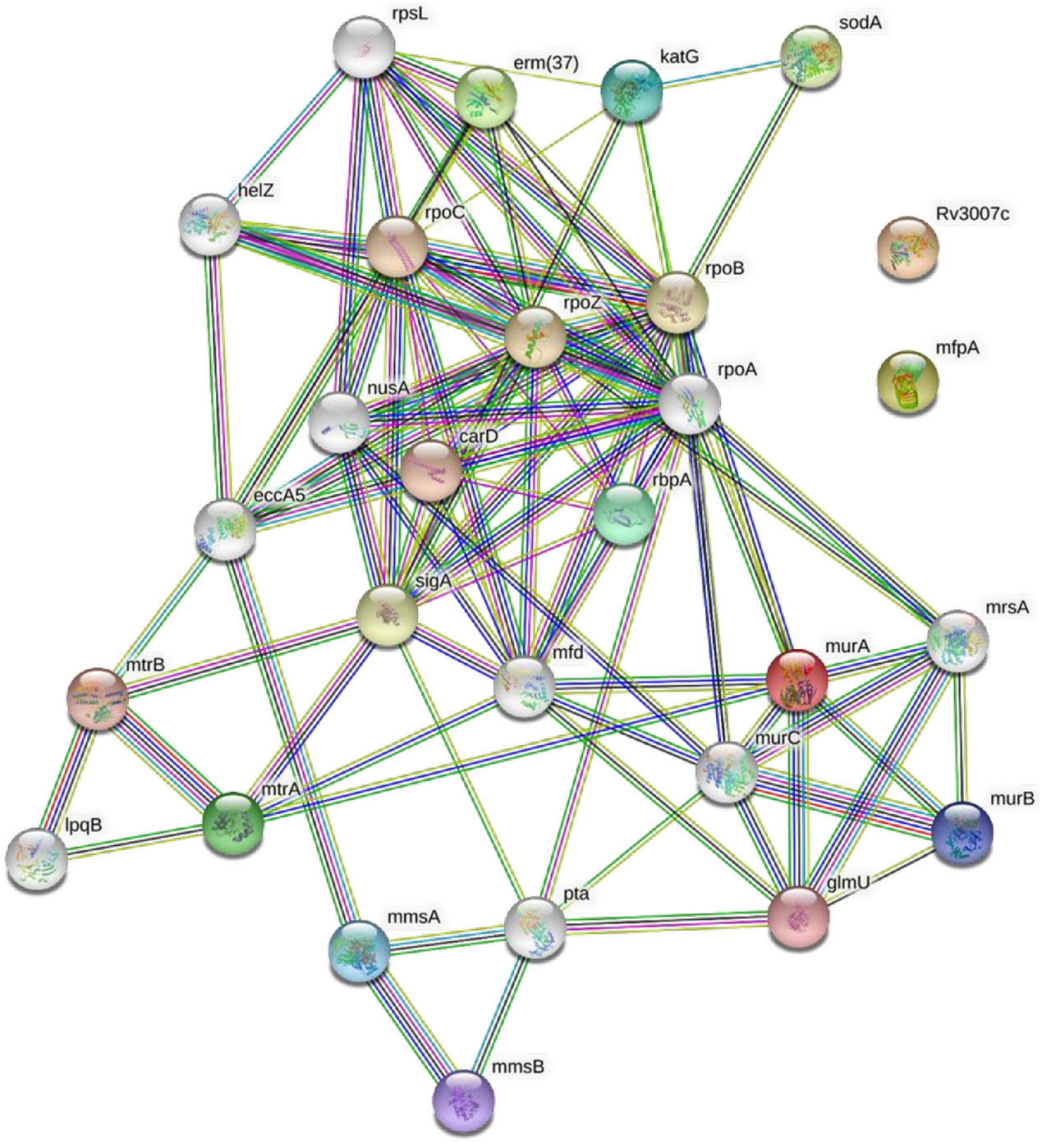
Protein–protein interaction network of AMR genes in MTB

The PPI network of MTB AMR genes was generated and analyzed using the STRING database, as depicted in Figure 1. In our analysis, we observed that four AMR proteins [mtrA, katG, erm (37) and murA] showed significant interactions with each other (*P*-value: < 1.0e–16). Consequently, mtrA and katG were selected for further docking analysis due to the availability of their crystal structure in PDB. To gain better insights, we further assessed the antigenicity and allergenicity of the proteins. However, none of the proteins, except murA, displayed allergenic characteristics (Table 3). It was determined that all proteins, except RbpA, exhibited antigenic properties, however, they do not show any allergenic reaction (except murA). All proteins were found as good drug targets based on antigenicity, allergenicity, pathway analysis and PPI analysis (Tables 2 and 3).

### 3.4 Identification of antimycobacterial phenolic compounds

To understand the antimycobacterial probabilities of selected phenolic compounds, we used the PASS server. In this analysis, many compounds were showing antimycobacterial activity toward MTB. Table 4 revealed the probability to be an antimicrobial and anti-mycobacterial drug candidate. A *P*-value of >0.5 was used to filter drug probabilities, PASS predicted that carvacrol, Limonene, p-Coumaric acid prenyl ester, 4-aminocinnamic acid, 4-nitrocinnamic acid were good inhibitors against MTB, showing *P*-value of >0.5. These five compounds are selected for docking analysis to confirm their antimycobacterial properties.

**Table 4.** List of phenolic compounds with antimicrobial and anti-mycobacterial activity

Class	Compounds	Smiles	Antibacterial activity	Anti-mycobacterial activity
Terpenoids	1,8-Cineole	CC1 (C2CCC (O1) (CC2)C)C	0.298	0.298
	Capsanthin	CC1=C (C (C[C@@H] (C1)O) (C)C)/C=C/C (=C/C=C/C (=C/C=C/C=C (\C)/C=C/C=C (\C)/C=C/C (=O)[C@@]2 (C[C@H] (CC2 (C)C)O)C)/C)/C	0.449	0.319
	Carvacrol	CC1=C (C=C (C=C1)C (C)C)O	0.319	0.540
	Carvone	CC1=CCC (CC1=O)C (=C)C	0.396	No
	Limonene	CC1=CCC (CC1)C (=C)C	0.405	0.610
	Manool	C[C@]12CCCC ([C@@H]1CCC (=C)[C@@H]2CC[C@] (C) (C=C)O) (C)C	0.425	No
	Nerol	CC (=CCC/C (=C\CO)/C)C	0.424	0.405
Flavonoids	Flavanone	C1C (OC2=CC=CC=C2C1=O)C3=CC=CC=C3	0.287	0.338
	Flavone	C1=CC=C (C=C1)C2=CC (=O)C3=CC=CC=C3O2	0.286	0.454
	Dihydroflavonol	C1=CC=C (C=C1)C2C (C (=O)C3=CC=CC=C3O2)O	0.303	0.266
	Flavonol	C1=CC=C (C=C1)C2=C (C (=O)C3=CC=CC=C3O2)O	0.331	0.376
	Flavan	C1CC2=CC=CC=C2OC1C3=CC=CC=C3	0.231	0.312
	Stilbene	C1=CC=C (C=C1)/C=C/C2=CC=CC=C2	0.253	0.421
	Isoflavonoid	CC1 (C=CC2=C (O1)C=C3C (=C2OC)C (=C (C (=O)O3)C4=CC5=C (C=C4)OCO5)O)C	0.352	0.210
	Neoflavonoid	CC (=O)OC1=C (C=C (C=C1OC)C2CC (=O)OC3=CC4=C (C=C23)OCO4)OC	0.219	No
Cinnamaldehyde	2-Bromo-3,4-dimethoxy-cinnamaldehyde	COC1=C (C (=C (C=C1)C=CC=O)Br)OC	0.187	0.203
	2-Nitro-4-methyl-cinnamaldehyde	CC1=CC (=C (C=C1)C=CC=O)[N+] (=O)[O-]	0.311	0.290
	2-Nitro-cinnamaldehyde	C1=CC (=CC=C1/C=C/C=O)[N+] (=O)[O-]	0.332	0.443
	3,4,6-Trimethoxycinnamaldehyde	COC1=CC (=C (C=C1C=CC=O)OC)OC	0.281	0.323
	3,4-Methylenedioxycinnamaldehyde	C1OC2=C (O1)C=C (C=C2)/C=C/C=O	0.233	No
	3-Methoxy-4-ethoxy-cinnamaldehyde	CCOC1=C (C=C (C=C1)/C=C/C=O)OC	0.206	0.330
	4-Dimethyl-amino-cinnamaldehyde	CN (C)C1=CC=C (C=C1)/C=C/C=O	0.264	0.250
	4-Methoxy-cinnamaldehyde	COC1=CC=C (C=C1)/C=C/C=O	0.260	0.319
	4-Nitro-cinnamaldehyde	C1=CC (=CC=C1/C=C/C=O)[N+] (=O)[O-]	0.332	0.443
	4-Dimethyl-amino-cinnamaldehyde	CN (C)C1=CC=C (C=C1)/C=C/C=O	0.264	0.250
	Caffeic_aldehyde	C1=CC (=C (C=C1/C=C/C=O)O)O	0.349	0.354
	Cinnamaldehyde	C1=CC=C (C=C1)/C=C/C=O	0.288	0.312
Cinnamic acids and derivatives	2-Coumaric_acid	C1=CC=C (C (=C1)/C=C/C (=O)O)O	0.355	0.489
	3,5-Diprenyl-4-coumaric_acid	CC (=CCC1=CC (=CC (=C1O)CC=C (C)C)/C=C/C (=O)O)C	0.408	0.383
	3-Coumaric_acid	C1=CC (=CC (=C1)O)/C=C/C (=O)O	0.330	0.458
	p-Coumaric acid prenyl ester	CC (=CCOC (=O)C=CC1=CC=C (C=C1)O)C	0.393	0.577
	4-Coumaric_acid	C1=CC (=CC=C1/C=C/C (=O)O)O	0.343	0.457
	4-Coumaryl alcohol	C1=CC (=CC=C1/C=C/CO)O	0.365	0.342
	4-Hydroxy cinnamyl alcohol diacetate	CC (=O)OCC=CC1=CC=C (C=C1)OC (=O)C	0.357	0.358
	4-Aminocinnamic_acid	C1=CC (=CC=C1/C=C/C (=O)O)N	0.372	0.546
	4-Chlorocinnamic_acid	C1=CC (=CC=C1/C=C/C (=O)O)Cl	0.261	No
	3-Nitrocinnamic_acid	C1=CC (=CC (=C1)[N+] (=O)[O-])/C=C/C (=O)O	0.333	0.434
	4-Nitrocinnamic_acid	C1=CC (=CC=C1/C=C/C (=O)O)[N+] (=O)[O-]	0.360	0.563
	3,4-Methylenedioxycinnamic_acid	C1OC2=C (O1)C=C (C=C2)/C=C/C (=O)O	0.263	0.287
	Benzylcinnamate	C1=CC=C (C=C1)COC (=O)/C=C/C2=CC=CC=C2	0.293	0.468
	Caffeic_acid	C1=CC (=C (C=C1/C=C/C (=O)O)O)O	0.358	0.486
	Ferulic_acid	COC1=C (C=CC (=C1)/C=C/C (=O)O)O	0.333	0.496
	Eugenol	COC1=C (C=CC (=C1)CC=C)O	0.325	0.332
	Eugenyl_laurate	CCCCCCCCCCCC (=O)OC1=C (C=C (C=C1)CC=C)OC	0.318	0.257
	Isoeugenol	C/C=C/C1=CC (=C (C=C1)O)OC	0.380	0.478
	Methyl_eugenol	COC1=C (C=C (C=C1)CC=C)OC	0.263	0.149
	Psoralen	C1=CC (=O)OC2=CC3=C (C=CO3)C=C21	0.328	0.334
Nitro phenol	4-Allyl-2-methoxy-5-nitrophenylacetate	CC (=O)Oc1cc (c (cc1OC)CC=C)N (=O)=O	0.371	0.314
	4-Allyl-2-methoxy-6-nitrophenol	COc1cc (cc (c1O)[N+] (=O)[O-])CC=C	0.316	0.314
	5-Allyl-3-nitrobenzene-1,2-diol	C=CCC1=CC (=C (C (=C1)O)O)[N+] (=O)[O-]	0.352	0.376
Phenyl ethers	Anethole	C/C=C/C1=CC=C (C=C1)OC	0.323	0.151
	Asarone	C/C=C/C1=CC (=C (C=C1OC)OC)OC	0.342	0.430
Phenol esters	2-Methoxy-4- (1-propenyl) phenylacetate	C/C=C\C1=CC (=C (C=C1)OC (=O)CC2=CC=CC=C2)OC	0.310	0.283
	4-Allyl-2-methoxyphenyl_acetate	CC (=O)OC1=C (C=C (C=C1)CC=C)OC	0.334	0.394
	Cinnamyl_benzoate	C1=CC=C (C=C1)/C=C/COC (=O)C2=CC=CC=C2	0.324	0.392

### 3.5 Molecular docking analysis

By molecular docking, the selected phenolic compounds were evaluated for their effectiveness against MTB AMR proteins. The selected compounds were docked with AMR proteins (mtrA and katG) to determine their docking potential and hydrogen bond interactions. As a result of docking, these phenolic compounds demonstrated a positive binding affinity and acceptable H-bond values with the amino acids of the AMR protein. As a result of the docking metrics described in Table 5, carvacrol, limonene, p-Coumaric acid prenyl ester, 4-aminocinnamic acid and 4-nitrocinnamic acid were identified as an inhibitor of MTB AMR proteins. Ethambutol is taken as a control in this study.

**Table 5.** Phenolic compounds and MTB target protein interaction analysis

S. no.	Compound name	mtrA			katG		
		Docking score	Number of H-bonds	Interacted amino acid residues	Docking score	Number of H-bonds	Interacted amino acid residues
1.	Carvacrol	–4.218	1	GLU126, ARG167	–6.161	2	ARG595, VAL507
2.	Limonene	–2.715	0	—	–5.075	0	—
3.	p-Coumaric acid prenyl ester	–3.105	1	ASP51	–3.587	1	R595
4.	4-Aminocinnamic acid	–3.053	1	ASP100, GOL2648	–2.167	1	R595
5.	4-Nitrocinnamic acid	–3.160	2	ASP160, ARG121, ARG122	–3.945	1	D513, D511, R595
6.	Ethambutol	–3.410	5	ASP100, ASP160, ARG119, ALA118	–1.739	4	VAL507, ASN508, ASH511, ASP513

#### 3.5.1 Active site and grid detection of mtrA and katG

Four active sites have been detected in KatG and two sites in mtrA protein. The active site with the highest site score was selected for ligand binding. KatG with a 1.091 site score and mtrA with a 0.976 site score value was selected for the docking process as the target site.

#### 3.5.2 Phenolic compounds against mtrA

**Figure 2.**
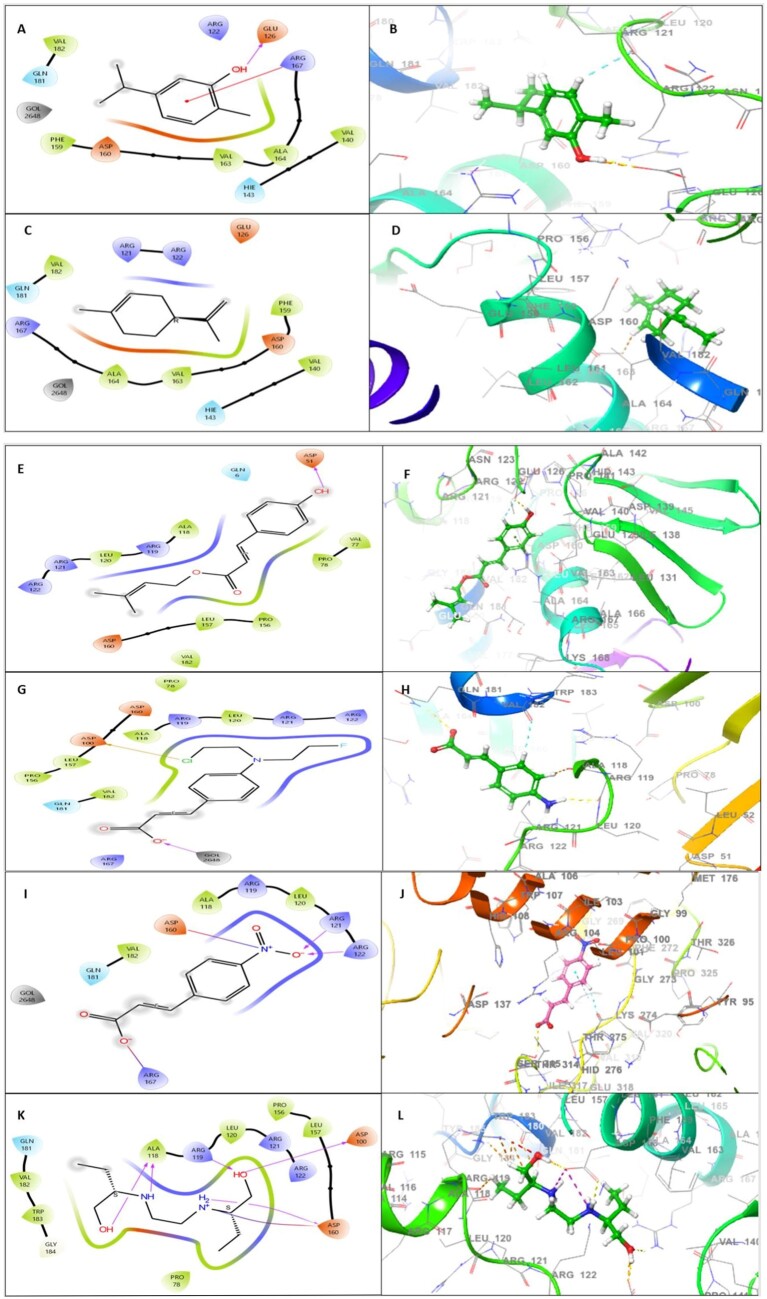
Molecular docking analysis: A 2D template is displayed to reveal the types of contacts formed between phenolic compounds and mtrA protein [(**A**) carvacrol, (**C**) Limonene, (**E**) p-Coumaric acid prenyl ester, (**G**) 4-aminocinnamic acid and (**I**) 4-nitrocinnamic acid, (**K**) Ethambutol]. 3D structure interaction analysis of phenolic compounds and mtrA protein [(**B**) carvacrol, (**D**) Limonene, (**F**) p-Coumaric acid prenyl ester, (**H**) 4-aminocinnamic acid and (**J**) 4-nitrocinnamic acid, (**L**) Ethambutol]

Carvacrol, limonene, p-Coumaric acid prenyl ester, 4-aminocinnamic acid and 4-nitrocinnamic acid were docked against mtrA protein. Carvacrol has the highest score –4.218 in reference to ethambutol (–3.410) with 1 H-bond interaction. In this docking complex, the ligand binds with mtrA protein residues GLU126 and ARG167 (Fig. 2). GLU126 forms a hydrogen bond backbone interaction with carvacrol. ARG167 showed π-cation interaction (Table 5, Fig. 2). These bonds are relatively strong and help to stabilize the complex formed between ligand and protein. Each additional hydrogen bond makes the interaction stronger. p-Coumaric acid prenyl ester, 4-aminocinnamic acid and 4-nitrocinnamic acid were also showing good docking scores –3.105, –3.053 and –3.160, respectively. Based on docking score and amino acid interaction, carvacrol has been identified as a potent inhibitor of the mtrA protein of MTB.

#### 3.5.3 Phenolic compound against katG

**Figure 3.**
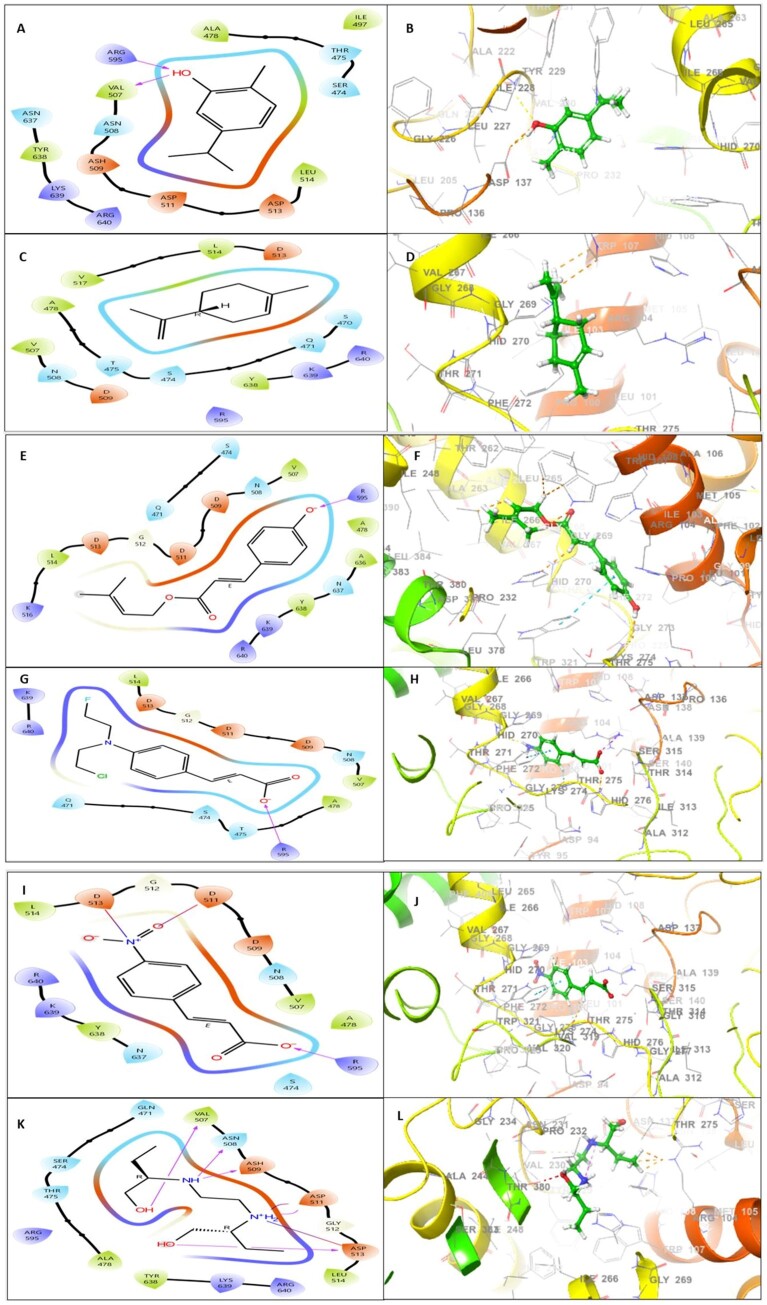
Molecular docking analysis: A 2D template is displayed to reveal the types of contacts formed between phenolic compounds and katG protein [(**A**) carvacrol, (**C**) Limonene, (**E**) p-Coumaric acid prenyl ester, (**G**) 4-aminocinnamic acid and (**I**) 4-nitrocinnamic acid, (**K**) Ethambutol]. 3D structure interaction analysis of phenolic compounds and katG protein [(**B**) carvacrol, (**D**) Limonene, (**F**) p-Coumaric acid prenyl ester, (**H**) 4-aminocinnamic acid and (**J**) 4-nitrocinnamic acid, (**L**) Ethambutol]

According to Table 5 and Figure 3, carvacrol has the highest docking score −6.161 with 2 H-bonds interaction, as its docking score is much higher than standard compound ethambutol (–1.739). The amino acid residues of katG such as ARG595 and VAL507 were implicated for their affinity toward carvacrol by forming two hydrogen bonds as shown in Figure 3A and B. This indicated carvacrol could be a good antimycobacterial candidate. Other phenolic compounds have good docking scores with respect to the standard compound ethambutol (–1.739) (Fig. 3K and L). Limonene has docking scores (–5.075) and showed interaction by no H-bond (Fig. 3C and D), p-Coumaric acid prenyl ester (–3.587) by 1 H-bond (R595) (Fig. 3E and F), 4-aminocinnamic acid (–2.167) by 1 H-bond (R595) (Fig. 3G and H), and 4-nitrocinnamic acid (–3.945) by 1 H-bond, ionic bond, and covalent bond with amino acid residue D513, D511, R595 (Fig. 3I and J). In this study, selected phenolic compounds showed good interaction with AMR proteins.

### 3.6 ADMET analysis

Drug candidates can be predicted based on their pharmacokinetic properties ADME and toxicity. ADME and toxicity analysis of selected phenolic compounds were shown in Tables 6 and 7. This process discards many drugs because of their poor pharmacokinetic properties. As a result of the ADME and toxicity profiling (Tables 6 and 7), selected phenolic compounds did not have any side effects associated with absorption except 4-aminocinnamic acid and 4-nitrocinnamic acid. Several ADMET properties associated with potential compounds for various models showed positive results that strongly supported the compounds' potential as drug candidates, including P-glycoprotein substrates, BBB penetration and gastrointestinal absorption. Based on the virtual screening, we identified Carvacrol as a hit anti-TB compound. Results revealed carvacrol as a leading inhibitor and it could serve as therapeutic inhibitors of MTB targets mtrA and katG.

**Table 6.** ADME analysis of selected phenolic compounds

Compound name	Adsorption					Distribution				Metabolism										Excretion	
	Caco-2 permeability	MDCK permeability	Pgp-inhibitor	Pgp-substrate	HIA	PPB	VD	BBB penetration	Fu	CYP1A2 inhibitor	CYP1A2 substrate	CYP2C19 inhibitor	CYP2C19 substrate	CYP2C9 inhibitor	CYP2C9 substrate	CYP2D6 inhibitor	CYP2D6 substrate	CYP3A4 inhibitor	CYP3A4 substrate	CL	T1/2
Carvacrol	–4.436	2.5e–05	0–0.1	0–0.1	0–0.1	93.128%	2.569	0.3–0.5	8.522%	0.7–0.9	0.7–0.9	0.3–0.5	0.3–0.5	0–0.1	0.3–0.5	0.3–0.5	0.3–0.5	0.3–0.5	0.3–0.5	11.335	0.671
Limonene	–4.320	1.9e–05	0–0.1	0–0.1	0–0.1	86.382%	3.373	0.9–1.0	9.244%	0.5–0.7	0.5–0.7	0.1–0.3	0.7–0.9	0.1–0.3	0.7–0.9	0–0.1	0.7–0.9	0–0.1	0.1–0.3	11.517	0.233
p-Couaric acid prenyl ester	–4.565	2.7e–05	0–0.1	0–0.1	0–0.1	96.307%	2.949	0.7–0.9	6.579%	0.9–1.0	0.1–0.3	0.9–1.0	0.1–0.3	0.7–0.9	0.9–1.0	0.3–0.5	0.5–0.7	0.1–0.3	0.1–0.3	15.739	0.903
4-aminocinnamic acid	–5.109	1.2e–05	0–0.1	0.7–0.9	0–0.1	73.557%	0.275	0.5–0.7	33.473%	0–0.1	0–0.1	0–0.1	0–0.1	0.3–0.5	0.1–0.3	0–0.1	0.1–0.3	0–0.1	0.1–0.3	7.177	0.753
4-nitrocinnamic acid	–4.691	6.6e–05	0–0.1	0–0.1	0–0.1	81.173%	0.318	0.1–0.3	8.050%	0.1–0.3	0–0.1	0–0.1	0–0.1	0–0.1	0.7–0.9	0–0.1	0.1–0.3	0–0.1	0.1–0.3	1.493	0.715

*Note*: *Adsorption*: Caco-2 Permeability (human colon adenocarcinoma cell lines Permeability): Empirical decision: > −5.15: excellent; otherwise: poor. MDCK Permeability (Madin−Darby Canine Kidney cells): A compound is considered to have a high passive MDCK permeability for a Papp > 20 × 10^–6^ cm/s, medium permeability for 2–20 × 10^–6^ cm/s, low permeability for <2 × 10^–6^cm/s. Pgp-inhibitor (P-glycoprotein): 0–0.3: excellent; 0.3–0.7: medium; 0.7–1.0: poor. Pgp-substrate (P-glycoprotein substrate): 0–0.3: excellent; 0.3–0.7: medium; 0.7–1.0: poor. HIA (Human intestinal absorption): 0–0.3: excellent; 0.3–0.7: medium; 0.7–1.0: poor. *Distribution*: PPB (Plasma protein binding): A compound is considered to have a proper PPB if it has a predicted value < 90%, and drugs with high protein-bound may have a low therapeutic index. VD (Volume Distribution): 0.04–20: excellent; otherwise: poor. BBB Penetration (blood–brain barrier): 0–0.3: excellent; 0.3–0.7: medium; 0.7–1.0: poor. Fu (The fraction unbound in plasms): >20%: High Fu; 5–20%: medium Fu; <5% low Fu. *Metabolism*: CYP 1A2/2C19/2C9/2D6/3A4 inhibitor and CYP 1A2/2C19/2C9/2D6/3A4 substrate: 0–0.3: excellent; 0.3–0.7: medium; 0.7–1.0: poor. The output value is the probability of being substrate/inhibitor, within the range of 0 to 1. *Excretion*: CL (The clearance of a drug): The unit of predicted CL penetration is ml/min/kg. >15 ml/min/kg: high clearance; 5–15 ml/min/kg: moderate clearance; <5 ml/min/kg: low clearance. T1/2 (The half-life of a drug): 0–0.3: excellent; 0.3–0.7: medium; 0.7–1.0: poor.

**Table 7.** Toxicity analysis of selected phenolic compounds

Compound name	Ames mutagenesis	Human Ether-à-go-go-Related Gene (hERG) Inhibition	Carcinogens	Acute oral Toxicity [log(1/(mol/kg))]
Carvacrol	No	Non-inhibitor	Non-carcinogen	1.63
Limonene	No	Non-inhibitor	Non-carcinogen	1.011
p-Coumaric acid prenyl ester	No	Non-inhibitor	Non-carcinogen	1.835
4-Aminocinnamic acid	No	Non-inhibitor	Carcinogen	2.247
4-Nitrocinnamic acid	Yes	Non-inhibitor	Carcinogen	1.101

*Note*: Significance value of Acute oral toxicity = 3.98 log(1/(mol/kg)) (Kutsarova *et al.*, 2021).

## 4 Conclusions

Only 10% of the symptoms are visible in MTB infection with the rest latent. Among infectious diseases, TB has caused the greatest number of deaths, and AMR is a particular reason. To develop new antimicrobial agents, the AMR targets must be identified to combat the rapid emergence of antimicrobial resistance among Gram-positive bacteria. The current study identified two AMR genes as targets (mtrA and katG) in MTB (Table 5). We focused on developing new antimycobacterial compounds against both targets because they are involved in metabolic pathways specific to pathogens. We found five phytochemicals that could be utilized in future TB clinical trials. Carvacrol has been identified with a significant docking score against both AMR genes (higher than ethambutol). We investigated the interaction of carvacrol with the amino acid residues of targets and found that it had the highest affinity for binding. Additional pharmacological and toxicological testing has been performed on antimycobacterial drug candidates as they could be used to treat TB (Table 6 and 7). Selected natural phenolic molecules possess a wide range of biological potential and pharmacological properties. This is the first study to show that they could combat AMR targets of MTB. According to the research output, carvacrol has the potential to be a powerful antimycobacterial drug. To gain a more understanding of targets and lead compounds, further, some more research work is warranted. Research in this field will continue to explore the identified compounds, optimize their properties, investigate combination therapies, utilize computational approaches, and advance toward clinical trials in the future. It is anticipated that these efforts will contribute to the development of more effective and targeted anti-TB agents for effective treatments as well as the ongoing fight against antimicrobial drug resistance.

## References

[vbad090-B1] Ballell L. et al (2005) New small-molecule synthetic antimycobacterials. Antimicrob. Agents Chemother., 49, 2153–2163. 15917508 10.1128/AAC.49.6.2153-2163.2005 PMC1140552

[vbad090-B2] Burley S.K. et al (2017) Protein data bank (PDB): the single global macromolecular structure archive. Methods Mol. Biol., 1607, 627–641. 28573592 10.1007/978-1-4939-7000-1_26 PMC5823500

[vbad090-B3] Camiade M. et al (2020) Antibiotic resistance patterns of *Pseudomonas* spp. isolated from faecal wastes in the environment and contaminated surface water. FEMS Microbiol. Ecol., 96, fiaa008. 31930390 10.1093/femsec/fiaa008

[vbad090-B4] Casadevall A. (2017) Antibodies to *Mycobacterium tuberculosis*. N. Engl. J. Med., 376, 283–285. 28099836 10.1056/NEJMcibr1613268

[vbad090-B5] Castro R.A.D. et al (2021) The within-host evolution of antimicrobial resistance in *Mycobacterium tuberculosis*. FEMS Microbiol. Rev., 45, fuaa071. 33320947 10.1093/femsre/fuaa071 PMC8371278

[vbad090-B6] Chakaya J. et al (2021) Global tuberculosis report 2020 – reflections on the global TB burden, treatment and prevention efforts. Int. J. Infect. Dis., 113 (Suppl. 1), S7–S12. 33716195 10.1016/j.ijid.2021.02.107 PMC8433257

[vbad090-B7] Chakaya J. et al (2022) The WHO global tuberculosis 2021 report – not so good news and turning the tide back to end TB. Int. J. Infect. Dis., 124, S26–S29. 35321845 10.1016/j.ijid.2022.03.011 PMC8934249

[vbad090-B8] Chen C. et al (2020) Detection of antimicrobial resistance using proteomics and the comprehensive antibiotic resistance database: a case study. Proteomics Clin. Appl., 14, e1800182. 31872964 10.1002/prca.201800182 PMC7378939

[vbad090-B9] Cheng F. et al (2012) admetSAR: a comprehensive source and free tool for assessment of chemical ADMET properties. J. Chem. Inf. Model., 52, 3099–3105. 23092397 10.1021/ci300367a

[vbad090-B10] Cragg G.M. et al (1997) Natural products in drug discovery and development. J. Nat. Prod., 60, 52–60. 9014353 10.1021/np9604893

[vbad090-B11] Eldeen I.M.S. , van Staden J. (2007) Antimycobacterial activity of some trees used in South African traditional medicine. South Afr. J. Bot., 73, 248–251.

[vbad090-B12] Farnsworth N.R. et al (1985) Medicinal plants in therapy. Bull. World Health Organ., 63, 965–981. 3879679 PMC2536466

[vbad090-B13] Hendriksen R.S. et al (2019) Using genomics to track global antimicrobial resistance. Front. Public Health, 7, 242. 31552211 10.3389/fpubh.2019.00242 PMC6737581

[vbad090-B14] Kanehisa M. et al (2022) KEGG mapping tools for uncovering hidden features in biological data. Protein Sci., 31, 47–53. 34423492 10.1002/pro.4172 PMC8740838

[vbad090-B15] Kumar B. et al (2022) Bioactive Phenolic Compounds from Indian Medicinal Plants for Pharmaceutical and Medical Aspects. IntechOpen, UK. doi:10.5772/intechopen.99672

[vbad090-B16] Kutsarova S. et al (2021) The QSAR toolbox automated read-across workflow for predicting acute oral toxicity: II. Verification and validation. Comput. Toxicol., 20, 100194. 10.1016/j.yrtph.2021.105015 34293429

[vbad090-B17] Ma Z. et al (2020) Screening and evaluation of *Mycobacterium tuberculosis* diagnostic antigens. Eur. J. Clin. Microbiol. Infect. Dis., 39, 1959–1970. 32548683 10.1007/s10096-020-03951-3

[vbad090-B18] Mazlun M.H. et al (2019) Phenolic compounds as promising drug candidates in tuberculosis therapy. Molecules, 24, 2449. 31277371 10.3390/molecules24132449 PMC6651284

[vbad090-B19] Nathanson S.D. et al (1977) Two-step separation of human peripheral blood monocytes on discontinuous density gradients of colloidal silica-polyvinylpyrrolidinone. J. Immunol. Methods, 18, 225–234. 201698 10.1016/0022-1759(77)90176-4

[vbad090-B20] Patridge E. et al (2016) An analysis of FDA-approved drugs: natural products and their derivatives. Drug Discov. Today, 21, 204–207. 25617672 10.1016/j.drudis.2015.01.009

[vbad090-B21] Pereira M. et al (2005) Drug resistance pattern of *Mycobacterium tuberculosis* in seropositive and seronegative HIV-TB patients in Pune, India. Indian J. Med. Res., 121, 235–239. 15817941

[vbad090-B22] Pontali E. et al (2019) Regimens to treat multidrug-resistant tuberculosis: past, present and future perspectives. Eur. Respir. Rev., 28, 190035. 31142549 10.1183/16000617.0035-2019 PMC9489134

[vbad090-B23] Prabhu S. et al (2017) Homology modeling and molecular docking studies on type II diabetes complications reduced PPARγ receptor with various ligand molecules. Biomed. Pharmacother., 92, 528–535. 28575810 10.1016/j.biopha.2017.05.077

[vbad090-B24] Prabhu S. et al (2021) Traditional uses, phytochemistry and pharmacology of *Bauhinia racemosa* Lam.: a comprehensive review. Futur. J. Pharm. Sci., 7, 101.

[vbad090-B25] Sakharkar K.R. et al (2004) A novel genomics approach for the identification of drug targets in pathogens, with special reference to *Pseudomonas aeruginosa*. In Silico Biol., 4, 355–360. 15724285

[vbad090-B26] Szklarczyk D. et al (2021) The STRING database in 2021: customizable protein–protein networks, and functional characterization of user-uploaded gene/measurement sets. Nucleic Acids Res., 49, D605–D612. 33237311 10.1093/nar/gkaa1074 PMC7779004

[vbad090-B27] Thomas M. et al (2017) Whole genome sequencing-based detection of antimicrobial resistance and virulence in non-typhoidal Salmonella enterica isolated from wildlife. Gut Pathog., 9, 66. 29201148 10.1186/s13099-017-0213-x PMC5697165

[vbad090-B28] Thomford N. et al (2018) Natural products for drug discovery in the 21st century: Innovations for novel drug discovery. IJMS, 19, 1578. 29799486 10.3390/ijms19061578 PMC6032166

[vbad090-B29] Tsaioun K. et al; The Alzheimer's Drug Discovery Foundation. (2009) ADDME – avoiding drug development mistakes early: central nervous system drug discovery perspective. BMC Neurol., 9, S1. 19534730 10.1186/1471-2377-9-S1-S1 PMC2697629

[vbad090-B30] Tyring S.K. (2012) Sinecatechins: effects on HPV-induced enzymes involved in inflammatory mediator generation. J. Clin. Aes. Dermatol., 5, 19–26. PMC3277090 22328955

[vbad090-B31] Veeresham C. (2012) Natural products derived from plants as a source of drugs. J. Adv. Pharm. Technol. & Res., 3, 200. 23378939 10.4103/2231-4040.104709 PMC3560124

[vbad090-B32] Xiong G. et al (2021) ADMETlab 2.0: an integrated online platform for accurate and comprehensive predictions of ADMET properties. Nucleic Acids Res., 49, W5–W14. 33893803 10.1093/nar/gkab255 PMC8262709

[vbad090-B33] Zaman K. (2010) Tuberculosis: a global health problem. J. Health Popul. Nutr., 28, 111–113. 20411672 10.3329/jhpn.v28i2.4879 PMC2980871

[vbad090-B34] Zhang J. , Tao A. (2015) Antigenicity, immunogenicity, allergenicity. Allergy Bioinf., 8, 175–186.

